# Total laparoscopic hysterectomy and bilateral salpingo-oophrectomy for stage 1 endometrial carcinoma under regional anaesthesia

**DOI:** 10.1016/j.gore.2023.101222

**Published:** 2023-06-08

**Authors:** Abdalla Fayyad, Mohammad Said, Moath Hasan, Mohammed Saleh

**Affiliations:** a Fayyad Centre for Minimal Access Surgery and Endometriosis, Amman, Jordan; b Royal College of Surgeons, Dublin, Ireland; c General Surgery, Khalidi Hospital, Amman, Jordan; d Abdel-Hady General Hospital, Amman, Jordan

**Keywords:** Laparoscopy, Hysterectomy, Uterine cancer, Epidural, Endometrial cancer

## Abstract

Total Laparoscopic hysterectomy is most commonly performed under general anesthesia. In elderly patients with severe medical co-morbidities and endometrial malignancy, laparoscopic surgery under general anesthesia can be hazardous. In such patients, regional anesthesia is safe, and can be the only option. We present a case of 75-year old women with severe lung fibrosis, chronic obstructive airway disease and heart failure who presented with severe post-menopausal bleeding and was diagnosed with endometrial carcinoma. She was considered unfit for general anesthesia. After multidisciplinary team meeting, the patient underwent total laparoscopic hysterectomy, bilateral salpingo-oophrectomy and peritoneal washings under regional anaesthesia. The procedure was successful and the patient was discharged 24 h later. In this case report, we demonstrate the anesthetic and surgical techniques for total laparoscopic hysterectomy under regional anesthesia. In the presence of dedicated multidisciplinary team, laparoscopic hysterectomy for endometrial carcinoma under regional anesthesia is safe and feasible.

## Highlights

• Surgery for endometrial carcinoma (CA) is usually performed under general anesthesia (GA).
• Laparoscopic hysterectomy & bilateral salpingo-oophrectomy (TLH & BSO) is best treatment.
• In the presence of severe co-existing medical conditions, GA may not be safe.
• We report endometrial CA treated under regional anesthesia for medical co-morbidities.
• We present tips for TLH & BSO under regional anesthesia.

## 1 Introduction

Hysterectomies are the most common gynaecological procedures performed for benign and malignant pathologies. In recent years, the laparoscopic approach to hysterectomy has gained popularity among appropriately trained laparoscopic surgeons (Moawad et al., 2018). Laparoscopic hysterectomy and bilateral salpingo-oophrectomy (BSO) is the preferred surgical approach for women with endometrial carcinoma (Van den Bosch and Mertens, 2016). Laparoscopic hysterectomy is typically performed under general anaesthesia (GA), as it provides control of the surgical pain and improves patient comfort with pneumoperitoneum and Trendelenburg position, along with providing a secure airway (Moawad et al., 2018). GA, however, is known to cause postoperative nausea and vomiting (PONV) along with sore throat, dental injury, and post-operative atelectasis (Moawad et al., 2018). In addition, GA can be unsuitable for patients with medical co morbidities such as chronic cardiopulmonary compromise. In this group of patients, regional anaesthesia (RA) allows safe conduct of surgery without potentially causing significant compromise of the patients’ cardiopulmonary functions (Vretzakis et al., 2014). Moawad et. al. reported a case of total laparoscopic hysterectomy (TLH) and bilateral salpingectomy under regional anaesthesia in a 51-year-old female for benign condition (fibroids and endometriosis) (Moawad et al., 2018). They showed that TLH was feasible under RA provided adequate counselling and effective communications exist. The patient in the case report above (Moawad et. al.) had a BMI of 22, and had no medical co-morbidities. In this case report, we describe a case of a total laparoscopic hysterectomy (TLH) and BSO under regional anesthesia for the treatment of endometrial adenocarcinoma in a patient who presented with severe post menopausal bleeding.

## 2 Case

A 75-year-old woman, para 5, with a body mass index (BMI) of 39, presented with post-menopausal bleeding for an extended period of time. Medical co-morbidities included type 2 diabetes mellitus, severe pulmonary fibrosis, hypertension, chronic obstructive pulmonary disease, heart failure, renal compromise and left carotid artery stenosis. MRI scan of the pelvis showed an endometrial mass with invasion of less than 50% of the myometrium with no evidence of pelvic lymphadenopathy. Outpatient endometrial sampling showed moderately differentiated endometroid adenocarcinoma. The provisional pre-operative staging was stage 1b endometroid adenocarcinoma of the endometrium. The patient underwent a pre-operative echocardiogram, which showed reduced ejection fraction of 30%. The lung function tests showed reduced vital lung capacity of 40% of normal, with reduced forced expiratory volume in the first second (FEV1) 0f 52% of the predicted volume. The patient was considered at high risk for general anaesthesia and systemic narcotics. Multidisciplinary team meeting recommended surgery in the form of TLH, BSO and peritoneal washings under regional anaesthesia due to the severe medical co-morbidities. We decided that pelvic lymphadenectomy was unnecessary, in line with the findings of the ASTEC trial (Kitchener et al., 2009) and the PORTEC trial (Creutzberg et al., 2000), and the need to expedite the surgery. An informed consent was obtained from the patient after thorough explanation of the technique of RA and the way surgery will be conducted. The patient was counselled about the dangers of general anesthesia including difficulty in performing successful extubation following surgery, the need for admission to intensive care unit, prolonged ventilation and death. The patient was also counselled about the risk of conversion to general anesthesia should she not tolerate pneumoperitoneum, or could not maintain her airway under regional anesthesia (RA).

The anaesthetic process started by administering 2 mg of Medazolam intravenously. The patient was positioned in the sitting position and under complete aseptic technique, and local anesthetic (1% Lidocaine) was administered subcutaneously. A combined spinal and epidural technique (CSE) was performed at the level of T10/T11 disc space. Consequently, Tuohy needle was inserted using loss of resistance to air technique. A spinal needle was inserted through the epidural needle. A mixture of 3 ml heavy Bupivacaine and 25 µg Fentanyl were injected intrathecally. Lastly, the spinal needle was removed, and the epidural catheter was inserted epidurally 3 cm inside the epidural space. The epidural top up was given as needed during the procedure with 0.25% Bupivicaine. Oxygen was administered through nasal cannula at a rate of 4 L per minute. The patient was hemodynamically stable throughout the procedure, and O2 saturation was around 97% throughout the procedure. Apart from intravenous Midazolam at induction, the patient was not given sedation nor opioids intravenously.

An orogastric tube was used to deflate the stomach to allow for safe entry of the Veress needle in Palmer’s point and avoid entry in a distended stomach. The orogastric tube was removed after successful port placement of the camera port. 1 g Tranexemic acid was given intravenously along with broad spectrum antibiotics. The patient was laid on her back with her legs positioned in hydraulic stirrups. Abdominal and vaginal cleaning was performed with Chlorhexidine, and Foley catheter inserted under aseptic technique. The cervix was dilated with Hegar dilators and V Care Plus uterine manipulator (CONMED, Largo, FL, USA) was inserted into the uterine cavity. A Palmer point Veress needle (Ethicon Inc., Somerville, NJ, USA) entry was performed, and pneumoperitoneum was established at 20 mm Hg pressure. A supraumbilical (4 cm above the umbilicus) 10 mm camera port was inserted under vision with the patient in flat position followed by two lateral ports at the level of the umbilicus (one 10 mm and one 5 mm ports) and one 5 mm port in the right iliac fossa inserted under vision after local anesthetic skin infiltration. The pneumoperitoneum pressure was then dropped to 12 mm Hg. The under surface of the right diaphragm (right diaphragmatic copula) was sprayed with 40 ml of 1% Lidocaine through a laparoscopic needle to reduce the shoulder pain from pneumoperitoneum during the procedure. The patient was tilted in the 15° head down Trendelenburg position. Peritoneal washings were obtained with a laparoscopic needle. The uterus, Fallopian tubes and ovaries were of normal size and appearance. A 30 degree laparoscope was used to allow for better visualisation of the pelvis due to the limited Trendelenburg position. This, along with thorough bowel preparation pre operatively and the use of uterine manipulator allowed for safe conduct of surgery despite the limited Trendelenburg position. The round ligaments were coagulated and cut bilaterally using Harmonic Ace + 7 advanced ultrasonic energy device (Ethicon Inc., Somerville, NJ, USA), and broad ligaments opened. Both ureters were identified, and the uterine artery was ligated bilaterally with absorbable 2/0 Vicryl sutures (Ethicon Inc., Somerville, NJ, USA) at its origin as they were leaving the anterior branch of the internal iliac artery when crossing the ureter. Both infundibulopelvic ligaments were coagulated and cut using the Harmonic device. The bladder was then dissected from the cervix using Harmonic device. The hysterectomy was then completed, separating the uterus from the parametrium and the uterosacral ligament bilaterally, and the vaginal cuff was then opened using the Harmonic device. The uterus, including the cervix tube and ovaries, were delivered en bloc through the vagina and sent to the pathology. The gas was evacuated, and the vaginal cuff was closed vaginally with continuous 2/0 Vicryl sutures after securing the angles of the vagina. Laparoscopic check was done at the end, and the fascia of the 10 mm ports were closed with 0 Vicryl sutures (Ethicon Inc., Somerville, NJ, USA). The duration of the procedure was 120 min, and the patient was relieved of the Trendelenburg position every 30 min (back to flat position) for 5 min to relieve pressure on the diaphragm and lungs and improve pulmonary compliance. The estimated blood loss was 100 ml.

The urinary catheter was removed the next morning, and the patient resumed normal voiding. The patient did not experience post-operative nausea nor vomiting, and had no postoperative complications. The patient was discharged 24 h after the procedure and had uneventful post-operative course. The patient’s experience of the procedure and regional anesthesia was positive, and reported that she would recommend it to a family or a friend. The histology showed a moderately differentiated grade 2 endometroid adenocarcinoma of the endometrium, which was stage 1b (invasion of less than 50% of the myometrium). The peritoneal washings were negative for malignant cells.

## 3 Discussion

Though laparoscopic surgeries have advanced in recent years, the approach to anaesthesia during these procedures has remained largely unchanged, with the majority of laparoscopic surgeries being performed under GA. The preference of GA for laparoscopic procedures has been largely due to its efficacy in controlling pain and reducing patient discomfort, particularly that caused by the pneumoperitoneum (Moawad et al., 2018). However, for certain patients who may be considered high risk for GA (like patients with pulmonary or cardiac comorbidities), providing the same procedure under RA is safer and has many benefits. RA has been known to reduce PONV, as well as postoperative pain and narcotic use (Shams et al., 2022). In addition, resumption of bowel motility has been shown to be quicker following RA. There are, however, other complications that can be caused by laparoscopic surgery under RA, such as shoulder pain, and the possibility of having to switch to GA during surgery. At the moment, the majority of laparoscopic surgeries performed under RA have been cholecystectomies, which cannot be compared to total hysterectomy due to the Trendelenburg positioning during hysterectomy, which decreases pulmonary compliance and has been known to cause greater discomfort for the patient (Singh et al., 2015). Della Corte et. al. recently reported a case series of five laparoscopic hysterectomies for benign conditions, and they showed that RA resulted in quicker recovery without compromising surgical results. It was noted that patients’ postoperative analgesics requirements were minimal with quick return of bowel function and post-operative mobility (Della Corte et al., 2022).

To our knowledge, this is the first case report of TLH for endometrial carcinoma performed under RA. A recent conference abstract reported a prospective cohort study of laparoscopic gynecological surgery for benign gynecological diseases. The study compared RA versus GA and found that the durations of surgery were comparable between the two groups, and no conversion to GA was required. The study showed that immediate postoperative pain was lower in the RA group, with faster resumption of bowel motility and patient’s mobilisation without significant pain reported during surgery due to the pneumoperitoneum (Della et al., 2022). The age of patients reported in the above case series were significantly younger than our patient. Furthermore, surgery was performed under RA as a patient choice for minimally invasive anesthesia rather than a significant risk attached to GA due to medical co-morbidities. The patient reported by Moawad et. al. (Moawad et al., 2018) and the group reported in the case series (Della Corte et al., 2022, Della et al., 2022) did not have significant medical co-morbidities. This is, in contrast to our patient, who suffered from serious medical co-morbidities, where surgery under GA would have been hazardous. In addition, the indication for hysterectomy in our case was a malignant condition causing severe post-menopausal bleeding.

Our case shows that TLH and BSO for endometrial carcinoma under RA is feasible and safe in elderly women with medical co-morbidities and increased BMI. Intraoperatively, the patient received adequate pain management using combined spinal epidural anesthesia (CSE) at the lower thoracic level, followed by the insertion of an epidural catheter. Intraoperatively, the injection of Lidocaine into the right copula of the diaphragm resulted in reduction of intraoperative shoulder pain. The procedure was done in the Trendelenburg position, set to 15 degrees tilt. The tilt was relieved every thirty minutes due to the pre-existing compromised pulmonary function. The 15° angle is important as it is the lowest possible degree of Trendelenburg which provides adequate access and visibility during surgery, while also minimising the reduction in pulmonary compliance (Mallick et al., 2015). We demonstrated that adequate pre operative bowel preparation, use of uterine manipulator, and 30 degree laparoscope, allow adequate access and visibility for TLH despite the limited tilt. Patients with normal lung function can tolerate a steeper degree of Trendelenburg position (up to 30 degrees). This, however, was not possible due to compromised ling function. Laparoscopic surgery can also cause pulmonary restriction due to the pneumoperitoneum during surgery. Reduction of the pneumoperitoneum pressure to 12 mmHg after laparoscopic ports insertion is important to reduce the risk of respiratory compromise. Although our routine practice is to perform vaginal cuff closure laparoscopically, the vaginal cuff was closed vaginally with continuous sutures after securing the angles. This was done to minimise the duration of the pneumoperitoneum and the Trendelenburg position during the procedure.

Although our team has long experience with performing vaginal hysterectomy, the patient was not suitable for this approach due to the poor vaginal access, high BMI, limited descent of the cervix on traction under anesthesia, and the need to remove the adnexae which can be challenging and hazardous, if done vaginally, in the presence of high BMI, and limited vaginal access. Open hysterectomy, on the other hand, would have been more hazardous due to the increased BMI, diabetes and possible worsening of lung expansion post operatively due to wound pain and increased risks of wound complications.

Our use of RA appears to have had many advantages; RA allowed for a safer anaesthetic option for our high-risk patient, for whom GA did not appear to be suitable due to the medical co-morbidities. It also led to quicker post-operative recovery, return to normal mobility and normal bowel movement post operatively, where the patient was discharged home 24 h after surgery. Furthermore, our approach eliminated the possibility of GA related side-effects, including admission to the intensive care unit, and reduction of PONV. In addition, many patients experience anxiety at the thought of being placed under GA, and the ability to ease that fear by opting for RA should not be understated.

In conclusion, TLH and BSO for endometrial carcinoma in the presence of high BMI and medical co-morbidities is safe and feasible under RA in the right multidisciplinary team setting with experienced anesthetic and surgical teams who maintain clear communication and intense collaboration. Our case supports the findings of Moawad et. al. that adequate ventilation and pain control can be achieved during laparoscopic hysterectomy under epidural anesthesia (Moawad et al., 2018). We also showed that adequate exposure for TLH can be achieved with limited Trendelenburg angle and pneumoperitoneum pressure. Furthermore, the muscle relaxation effect of epidural anesthesia was adequate for safe performance of the surgery, without the need for profound muscle relaxation. Not only does the option of RA allow patients more choice in anesthesia during surgery, but can be the only anesthetic option available to patients with advanced medical co-morbidities who need to undergo TLH for endometrial carcinoma.

## Precis

Description of technique of total laparoscopic hysterectomy and bilateral salpengo-oophrectomy under regional anesthesia for a woman with post-menopausal bleeding and endometrial carcinoma with significant medical co-morbidities that prohibit general anesthesia.

## Ethical approval

None required**.** Patient consent was obtained for publishing the case report.

## Declaration of Competing Interest

The authors declare that they have no known competing financial interests or personal relationships that could have appeared to influence the work reported in this paper.
